# Higher Order Thinking by Setting and Debriefing Tasks in Dutch Geography Lessons

**DOI:** 10.3390/ejihpe12010002

**Published:** 2021-12-30

**Authors:** Uwe Krause, Tine Béneker, Jan van rtwijk

**Affiliations:** 1 Faculty of Geosciences, Utrecht University, 3584 CB Utrecht, The Netherlands; t.beneker@uu.nl; 2 Department of Geography Education, Fontys University of Applied Sciences Tilburg, 5022 DM Tilburg, The Netherlands; 3 Faculty of Social and Behavioural Sciences, Utrecht University, 3584 CS Utrecht, The Netherlands; j.vantartwijk@uu.nl

**Keywords:** tasks, geography, higher order thinking, recontextualization, geography didactics, powerful knowledge, cumulative learning, curriculum context

## Abstract

Tasks are a powerful instrument for geography teachers, as they let students engage with the subject. To advance the cumulative learning of students, teachers have to make sure that students learn how to deal with complex and abstract knowledge structures. In the Netherlands, teachers face a dilemma when it comes to task setting: the intended curriculum aims for a considerable part at (parts of) higher order thinking, whereas the high-stakes exams have a clear focus on the use of thinking strategies. This paper explores the task setting and debriefing of Dutch geography teachers by analyzing twenty-three videotaped lessons in upper secondary education by using the Geography Task Categorization Framework. The results show that Dutch teachers mostly rely on textbooks when setting tasks. The focus lies on reproduction and the use of thinking strategies. Tasks aiming at (parts of) higher order thinking are barely used. Furthermore, teachers use tasks from previous high-stakes exams already used in an early stage of upper secondary education. In the debriefing of tasks, teachers move from simple and concrete to complex and abstract knowledge and vice versa. However, most of these movements aim at simplifying knowledge structures. In the observed lessons, curriculum aims at the level of (parts of) higher order thinking are not achieved. The evaluative rules as set by the high-stakes exams and the type of tasks offered by textbooks seem to be dominant.

## 1. Introduction

Tasks let students interact with the content of the subject [1]. Accordingly, tasks have a prominent place in geography lessons. Earlier research shows that in Dutch upper secondary geography lessons, working on tasks and debriefing accounted for 38% of the lesson time [2]. An important aspect of setting tasks a teacher must consider is the development of general and subject specific competences [3]. “Cumulative learning is at the heart of education” [4] (p. 199), and students should increasingly engage with complex knowledge structures and the abstract nature of knowledge, which Winch refers to as the “epistemic ascent” [5]. Fostered by higher order thinking tasks stimulating critical and creative reasoning and showing that knowledge can be fallible, this epistemic ascent ideally leads to knowledge of high epistemic quality, which is an essential characteristic of powerful knowledge [6,7]. However, as Vernon [8] pointed out, epistemic ascent is not necessarily linear as, for example, there is an annual increase in complexity and abstraction. On the contrary, the learning process should be understood as constantly moving between the very concrete and the very abstract. Furthermore, there is no guaranteed pedagogical approach towards high epistemic quality, as in the classroom setting the teaching is always mediated by factors and characteristics beyond the control of the teacher [9].

Thus, tasks are key for teachers to fulfil their role as curriculum makers, which is building a curriculum of student’s engagement with the subject [9]. Setting and debriefing tasks by teachers can be understood best as “a reflective process of transformation and/or recontextualization of knowledge” [10] (p. 209). This means that teachers have to choose tasks consciously, in order to achieve aims of subject-specific learning. These aims can be an interpretation of subject-specific knowledge by the teacher, or an interpretation of the intended curriculum as described in curriculum documents. The role of the teachers in this process of recontextualization, or in other words didactical reconstruction, often is underestimated [11]. According to Van den Akker [12], tasks are part of the “curriculum-in-action” (p. 3). They are the link between the intended curriculum, which is laid down in vision documents and the formal written curriculum, and the attained curriculum, which refers to the learning experiences and results by the students (see also [13]). 

In the Dutch context, teachers face a dilemma between the intended curriculum as specified in curriculum aims, and the evaluative rules of the curriculum context [14]. With evaluative rules we refer to all procedures which transmit criteria the learner has to fulfil in order to produce what is considered to be a “legitimate text” in respect of knowledge structure and adequate language [15]. The Dutch curriculum aims refer for a considerable part to higher order thinking or parts of it [14]. The high-stakes exams, however, do not encompass higher order thinking tasks, and also in geography textbooks for upper secondary, higher order thinking tasks are scarce [16,17]. Evaluation rules emphasize, among other things, specific formulations such as cause-and-effect relationships. The knowledge to be acquired by students is described and prescribed in detail in respect of substantial concepts and generalizations/rules. Furthermore, teachers are held accountable for the achievement and quality of the average expected learning output [14]. This paper aims at exploring how Dutch teachers in this situation of ambiguity recontextualize geographical knowledge by their task setting and debriefing in upper secondary education.

## 2. Theoretical Framework

This research builds on theoretical insights of the educational sociologists Basil Bernstein [15,18,19] and Karl Maton [4], which are at the base of the conceptualization of “powerful knowledge” [20]. This concept has had a strong influence on the debate in geography education in various countries including The Netherlands [21]. We will first outline their thoughts to better understand the role of the teacher in the recontextualizing process in respect of geography tasks. Subsequently we will describe the Geography Task Categorization Framework as a means to analyze cognitive processes fostered by tasks. We then describe the Dutch curriculum context in respect of setting tasks. Finally, we focus on aspects teachers have to consider while task setting in geography lessons in order to promote subject-specific competence development. 

### 2.1. The Context of the Recontextualization Process in Respect of Setting and Debriefing Tasks

In Bernstein’s view [15] (p. 25), education can be seen as a “pedagogic device”, which transforms knowledge into pedagogic communication. Communication within that device (i.e., the type of tasks in our research) is regulated by two main principles of control [15,18]: classification and framing. Classification is about the content selection in respect of the curriculum: “the what”. It encompasses declarative as well as procedural knowledge, and acts through recognition rules, which regulate the level of complexity and abstraction. Framing relates to the pedagogy and ways of transmission: “the how”. It works through realization rules. These rules determine “how we put meaning together and how we make them public” [15] (p. 17). By sequencing, pacing and assessment, the acquisition of knowledge as well as expected outcomes and how they are communicated are controlled. Classification and framing always work together and regulate the three subfields of the pedagogic device. First is the primary context, which is the field of scientific knowledge production. Second is the recontextualizing field, where different agents such as departments of the state, universities of education or educational media transfer the educational discourse from the primary to the secondary context. Third is the secondary context, which is the field of pedagogic practice taking place in schools at various levels (teachers, departments, direction), and where the educational discourse is reproduced selectively. Thus, before its realization at school, the educational discourse has undergone two recontextualizations (see also [4] (p. 90)), which have to be understood as a principle, “which selectively appropriates, relocates, refocuses, and relates other discourses to constitute its own order” [15] (p. 33). 

In the view of Bernstein, the type of tasks used during lessons “enable that acquirer to construct the expected legitimate text … In this system a text is anything which attracts evaluation” [15] (p. 18). What type of tasks are required, and what the legitimate text is, is determined by recontextualizing rules and evaluative rules. Recontextualizing rules direct the pedagogic discourse, which is produced by official institutions, such as ministries or the school inspectorate) and pedagogic agencies, such as departments of education at universities or educational publishers. These rules compose the “thinkable”, the official knowledge [15]. Evaluative rules construct any pedagogic structure, because their purpose is to transmit criteria for evaluation. According to Bernstein [15] (p. 18), evaluation “condenses into itself the pedagogic code and its classification and framing procedures, and the relationships of power and control that have produced these procedures”. This makes the role of the teacher key when it comes to task setting in geography lessons, as the production of the legitimate text is not something reproduced mechanically, but is produced actively by the interactional practice between student and teacher [15]. To describe the teachers’ competences and behavior, Bernstein [22] uses the concept of repertoire. He defines repertoire as a “set of strategies any one individual possesses and their analogical potential for contextual transfer” [22] (p. 159). The repertoire of the teacher depends on his/her reservoir (the secondary context the teacher is functioning in) and access to the vertical context (scientific knowledge) and on the curriculum context. Hence, the choices made in the curriculum context become visible in how teachers set tasks during geography lessons.

### 2.2. Cognitive Processes Fostered by Tasks: Geography Task Categorization Framework

**Figure 1:**
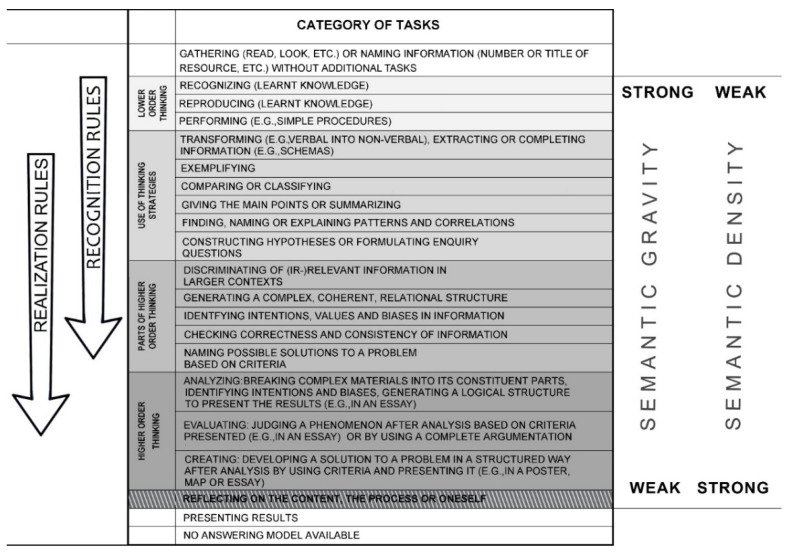
Geography Task Categorization Framework—the arrows indicate the increase of importance of recognition and realization rules (adapted from [16])—including the concept of semantic gravity and density of Maton [4].

When it comes to competence development, categorizations of tasks define increasing levels of difficulty with respect to actions expected from the student. They refer to the ways existing knowledge has to be applied and how complex the data/resources are that have to be manipulated [23]. In categorizations of geography tasks, often a distinction is made between lower and higher [24,25,26], or lower, middle and higher order thinking [27,28]. However, lower order thinking is not always defined in the same way: it can be restricted to the reproduction of knowledge, but it can also encompass the application of acquired knowledge. In order to overcome the simple dichotomy of lower versus higher order thinking based on Bloom’s revised taxonomy [29], the Geography Task Categorization Framework (GTCF) [16] was developed (see Figure 1), which displays meaningful variations in respect of cognitive processes. The instrument considers recognition as well as realization rules, and distinguishes four elements on the continuum between lower and higher order thinking: (1) the provision of new contexts, (2) the complexity of the contexts (resources) and the use of criteria, (3) independent reorganization of resources, use of criteria and representation by the learner, and (4) metacognition. 

The instrument allows us to identify how tasks foster powerful knowledge. Powerful knowledge, a concept introduced by Young [20], states that all learners from all backgrounds should have access to disciplinary, subject-specific knowledge. This knowledge, transcending the limits of the students’ own experience, enables students to think in new and critical ways [30]. According to Béneker [21], the five aspects of powerful knowledge are: (1) factual or concrete geographical knowledge, (2) conceptual and theoretical knowledge, (3) systematic knowledge, which stems from the combination of the first two types of knowledge,(4) knowledge and language of societal debates, which refers to problem solving and supporting own standpoints by argumentation, and (5) knowledge of knowledge, which enables students to check correctness and bias of information, sensitizes them to discourses within the subject discipline and introduces them to ways of knowledge production. The five levels of the GTCF display an increase in abstraction, which according to Maton [4] (p. 180) are “central to the recontextualization of knowledge”. 

Maton [4] emphasizes the role of language in this process, and distinguishes between semantic gravity and semantic density, which both have to be seen as a continuum (see Figure 1). Semantic gravity refers to the extent to which meaning is related to the context, in other words, how concrete or how abstract the knowledge is. This refers to recognition rules. Tasks with a strong semantic gravity stand for concrete knowledge in well-described contexts. There is generally one correct answer, the focus lies more on the body of knowledge, which already exists or just has been achieved, and has to be recalled. A weak semantic gravity refers to complex, abstract and generalized ideas, where Bernstein’s “discursive gap” [15] (p. 30) becomes visible: criteria have to be chosen and accounted for, and this leads to more possible “correct” solutions. Semantic density stands for the extent of condensation of meanings in language, and refers to realization rules and expected outcomes. In respect of tasks, semantic density becomes visible in students’ answers. A strong semantic density relates to complex conceptual ideas, which have to be articulated. A weak semantic density points at one, concrete meaning of a (substantial) concept. Debriefing questions help to strengthen or to weaken the semantic density. While strengthening semantic density, debriefing questions move from concrete facts or events to overarching concepts, whereas debriefing questions helping to simplify complex and abstract concepts are weakening the semantic density [4] (pp. 211–212).

### 2.3. Tasks and the Development of Subject-Specific Competences 

To realize the students’ cumulative learning, the teacher has to have a clear idea about students’ prior knowledge and the curriculum aims in order to close the gap between the intended aims and learning objectives to be accomplished [31]. Task setting is a very complex activity [3], in which next to the subject-specific competence development the teacher has to consider how tasks can motivate and engage the students [32,33], how to address the heterogeneity of the students [34,35], how to structure the learning process [36] and formulate the tasks [37], how to foster individual as well as cooperative learning [38,39], and how materials can promote a broad scale of subject-specific skills [40]. A key aspect for competence development is the debriefing of tasks. Nichols [41] distinguishes here between the typical question-and-answer episode, which is characterized by closed, lower order questions focusing on recall of facts and simple ideas, and debriefing episodes, which encompass open questions aiming at correlations, reasoning, constructing hypotheses and justifying. In the latter case there is more emphasis on students’ contributions on what and how they have learnt, and possibilities for feedback, which has a positive impact on learning results [42]. An important aspect in the debriefing is argumentation to come to evidence-based answers [43]. However, empirical research in the German context shows that, on average, the subject-specific argumentation of both secondary school students and university students is poor [44].

As Maton [4] (p. 44) points out, the ways of working in the classroom shape by their routines what he calls the “rules of the game”, by which he means what has to be understood as the legitimate text. So not only in the task setting, but also in the debriefing of the tasks, becomes visible what can be considered as the “dominant basis of achievement” [4] (p. 54). He suggests to use the concepts of semantic gravity and density in order to reveal how knowledge practices facilitate or constrain cumulative learning [4]. Students have to learn to move between concrete and abstract knowledge, as these “movements in semantic gravity provide a necessary (though not sufficient) condition for the decontextualization and recontextualization of cumulative knowledge-building and learning” [4] (p. 201). Mastering semantic gravity and density, according to Maton ([4] see also [8]), is a key aspect of powerful knowledge, as it enables students to use factual knowledge and connect abstract, conceptual knowledge to the concrete but complex reality, and by doing so empowers students to take part in societal debates. This means that for our research it is not only necessary to explore what types of tasks are set by teachers in geography lessons, but also how these tasks are debriefed.

## 3. The Dutch Context in Respect of Setting and Debriefing Tasks

The application of the instrument for analyzing curriculum aims showed that the Dutch curriculum, besides the acquisition of a body of conceptual and factual knowledge, stresses the importance of systematic knowledge. More than half of the curriculum aims refer to this type of knowledge. Nevertheless, a considerable part, i.e., one-fifth of the curriculum aims, refers to (parts of higher) order thinking, especially evaluation [14,17]. To understand the Dutch curriculum context, it is important to know that there is a difference in the program between curriculum aims, which are assessed in the high-stakes exam, and aims, which are assessed only in a school exam and not in the high-stakes exam. The Dutch high-stakes exam consists of around thirty questions about eight topics. It clearly focuses on systematic knowledge. The assessment of the school exam lies in the hands of the teacher. Here the topics are among others the global food issue and climate issues, which should prepare students to participate in societal debates, and therefore curriculum goals especially aim at (parts of) higher order thinking [14,17].

However, school and high-stakes exams cannot be seen as separated from each other. There are control mechanisms, such as the rule that the marks for the school exam and the high-stakes exam should not deviate too much [45]. This leads to a pre-shadowing effect of the high-stakes exam on the school exams [26]. This pre-shadowing effect also becomes visible in the tasks that textbooks offer about the global food issue and climate issue [17]. The textbook tasks mirror the tasks in high-stakes exams in respect of cognitive processes. An analysis of textbook tasks about the global food issue and climate issue in the three textbooks used in Dutch geography education showed that approximately one-third of the tasks aimed at lower order thinking and nearly two-thirds at the use of thinking strategies. Only a few tasks (7.7% for the global food issue and 5.1% for the climate issue) could be categorized as (parts of) higher order thinking, metacognition or presentation of results [16,17]. However, these aspects are key for the epistemic ascent of students and important components of powerful knowledge, and also part of the intended curriculum. So, how teachers pay attention to higher order thinking when setting and debriefing tasks is an important question.

## 4. Research Question

Setting tasks in geography lessons is key for the development of powerful knowledge of students, and can be understood as a form of recontextualization of the intended curriculum by the teacher. As pointed out, in the Dutch context the teacher has to face a dilemma. The curriculum aims for upper secondary, which is the pre-university level for students between 16 and 18 years old, aim at the combination of all cognitive levels, including higher order thinking. In the high-stakes exam and textbooks, tasks aiming at lower order thinking and the use of thinking strategies are dominant. Furthermore, evaluative rules reinforce the type of tasks used in high-stakes exams. To explore the recontextualization by the task setting of teachers, our research question is:

*To what extent are the tasks set in Dutch geography lessons for upper secondary in line with the intended curriculum?*

We formulated two questions that should help to answer the research question:

*What cognitive processes do the tasks set during geography lessons foster?*

The type of tasks used in geography lessons show how teachers recontextualize the discipline of geography in their lessons. By answering this question, we get a clearer picture of how the tasks meet the cognitive processes aimed at by the curriculum.

*How are the results of the tasks discussed during the geography lessons?*

The way of debriefing (together with the type of tasks set) can be seen as the routine, which reveals what can be considered as legitimate text. The semantic density, and especially the movement between the two ends of this continuum, indicates to what extent the debriefing contributes to cumulative learning, to the development of powerful knowledge, and is in line with the curriculum aims.

## 5. Methodology

Between October and December 2018 twenty-five lessons were observed and video-recorded by a group of five master’s students of geography education. This research is based on twenty-three of these video-recordings, as two of the observed lessons were not completely recorded. The teachers were contacted by the master’s students. The schools of the teachers are located in the south of the Netherlands. All lessons observed took place at the highest level of education, *voortgezet wetenschappelijk onderwijs (vwo)*, which gives direct access to research universities. This means that the results are comparable in an international context [46]. The teachers were asked to teach an ordinary lesson. After the lesson, the teachers and 1–3 students who participated in the lesson confirmed that the observed lesson was comparable with how lessons were generally taught. All teachers and students participating were informed that the data would be used for research purposes, that participation was voluntary, and gave their consent. The use of the data was approved by an ethical commission of Utrecht University.

Based on the filmed lessons, we recorded:

- If, how long (in seconds), and how many tasks were used,
- If, what and how many questions were asked by the teacher,
- If the tasks derived from the textbook (and which one) or not (selected by the teacher from other resources or developed by the teachers themselves), or originated from high-stakes exams from recent years,
- If the students worked individually, in pairs, in groups or as a whole class,
- If the students worked on tasks and if the tasks were debriefed.

All tasks used in the lessons were categorized by two experts on subject pedagogy by using the Geography Task Categorization Framework [16] considering the answering models for the tasks when possible. The interrater reliability was sufficient (Cohen’s Kappa 0.84). We found disagreement when the answer to be given was derived from the continuous text in the textbook, and therefore should be considered as reproduction. Subsequently all items were discussed in order to establish full interrater agreement (see [47]).

Next, all questions asked by the teachers during the lessons, 489 in total, were transcribed and coded by two experts on subject pedagogy by using the Geography Task Categorization Framework. The interrater reliability was high (Cohen’s Kappa 0.95). For 24 questions, disagreement mostly occurred when the questions related to earlier acquired knowledge. These issues were discussed in order to achieve full agreement at the end.

The categorizations of tasks and debriefing questions were analyzed in relation to each other, the topic (physical or human geography), the origins of the task (textbook, former high-stakes exams, other), the class (10th, 11th or 12th grade), and according to characteristics of teachers including age, gender, experience, education (master’s degree of a research university of a university of applied sciences), work field (only upper secondary, upper and lower secondary mixed). Furthermore, it was indicated when the teacher referred with his or her question or remark to the high-stakes exam. In these cases we analyzed if the remarks correlated with the characteristics of teachers, the type and the origins of the task, or the topic.

We used correlation analysis in IBM SPSS 23.0 to check the relation of variables and *t*-test or ANOVA to test group differences based on the mean score of the research variable. As we performed multiple tests, we applied a Bonferroni correction where necessary.

## 6. Results

The average lesson time of the 23 observed lessons (see Table 1) was 46:17 min, of which 42:09 min were effective. The rest of the lessons were made up of organizational issues, or the teacher came late, etc. Of these, 42:09 min (see Table 1) tasks accounted for 53% (SD 18.32).

The lowest percentage of time spent on tasks in the observed lessons was 18%; the highest was 95% (SD 18.36). On average, students worked on tasks during 27% of the time of a lesson. In 22% of the time, the outcomes of tasks were debriefed, and in 5% of the time teachers chose an approach of letting students perform the tasks while discussing the answers with them at the same time. We checked the group differences with independent sample *t*-tests for two groups and we used analysis of variances for more than two groups. There were no statistically significant differences in respect of gender, age, experience, work field (only upper secondary, or lower and upper secondary), education of the teacher (university or university of applied sciences), or in respect of class (4th, 5th or 6th grade), the topic (physical or human geography), or the origin of the task (textbook, high-stakes exams, other materials). 

Of the 238 tasks set during the observed lessons, 80%, derived from the textbook, 11% of the tasks derived from other materials or had been developed by the teacher, and 8.7% of the tasks derived from previous high-stakes exams. Strikingly, in three of the four cases where teachers used exam tasks, these were not used in the final year. A one-way ANOVA between groups was performed to compare the number of tasks set in a lesson with the origin of the tasks. There was a statistically significant difference between the groups *F*(2, 26) = 11.96, *p* < 0.001. After applying a Bonferroni correction, textbook tasks (*M* 12.38, *SD* 5.63) correlated significantly with more tasks set during a lesson compared to high-stakes exam tasks (*M* 5.25, *SD* 3.4) and other tasks (*M* 2.57, *SD* 1.81). 

On average, teachers used 10.5 tasks per lesson (see Table 2). The standard deviation (6.49), however, indicates considerable differences: three teachers applied only one or two tasks, while five teachers applied more than 15 tasks.

About one-third of the tasks set by the teachers can be labeled as lower order thinking (LOT) and focused mainly on the reproduction of knowledge. Two-thirds of the tasks aimed at the use of thinking strategies (UTS), and merely on patterns and correlations, the manipulation and extraction of information from resources, and comparing and classifying. Only very few tasks could be categorized as parts of higher thinking (PHOT) or focused on metacognition. Tasks of the category higher order thinking (HOT) were absent.

In order to detect differences between groups who used less or more tasks and the type of tasks they used, we created groups by applying the nested means approach [48]. First, the mean value for all teachers was calculated and then again the mean value for the lower and upper group. This led to four groups: 1–3 tasks, 4–10 tasks, 11–15 tasks and 16–20 tasks. A one-way ANOVA between groups was performed to compare the impact of the number of tasks set in a lesson on the type of tasks. There was a statistically significant difference between the groups *F*(3, 22) = 22.95, *p* < 0.001 in the use of LOT tasks. After a Bonferroni correction, the first group (*M* 0.25, *SD* 0.5) and the second group (*M* 0.67, *SD* 0.82) used significantly fewer lower order thinking tasks than the third group (*M* 4.88, *SD* 1.36) and the fourth group (*M* 5.8, *SD* 2.17). Furthermore, there was a statistically significant difference between the groups in respect of the use of UTS tasks *F*(3, 22) = 24.29, *p* < 0.001. Additionally, after the Bonferroni correction, the first group (*M* 0.75, *SD* 0.5) and the second group (*M* 6.67, *SD* 2.58) used significantly fewer thinking strategy tasks than the third group (*M* 8.63, *SD* 2.07) and the fourth group (*M* 11.8, *SD* 2.28). Finally, there was a significant difference between the groups in respect of the use of PHOT tasks *F*(3, 22) = 5.23, *p* < 0.01. Only the first group used this type of task (*M* 0.5, *SD* 0.58), and also after the Bonferroni correction, this difference was statistically significant.

Furthermore, a one-way ANOVA between groups showed a statistically significance between the origins of the task and the type of tasks: for LOT tasks it was *F*(2, 26) = 12.47, *p* < 0.001, and for UTS tasks *F*(2, 26) = 7.83, *p* < 0.01. The post hoc Bonferroni test displayed that lower order thinking tasks occurred significantly more often when textbook tasks (*M* 4.31, *SD* 2.5) were set compared to when high-stakes exam tasks (*M* 0.25, *SD* 0.5) or other tasks (*M* 0.43, *SD* 0.79) were set. UTS tasks were applied less often when teachers used their own materials (*M* 1.71, *SD* 1.5) and compared to textbook tasks (*M* 7.91, *SD* 4.12) the difference was statistically significant. A more detailed analysis shows that the textbook tasks set correlated significantly more with recognition and transforming, extracting or completing information. When own tasks were set, they correlated significantly more with summarizing and significantly less with recognizing, describing and naming of patterns and correlations.

Regarding the debriefing, we observed that in 17 of the 23 lessons a debriefing of tasks took place, sometimes combined with the performing of tasks at the same time. In these 17 lessons, teachers spent 36% (*SD* 20.9) of the effective lesson time on debriefing. However, there were considerable differences, ranging between 5% and up to 60% of the lesson time. 

In total 96, tasks were debriefed (see Table 3), on average 5.65 tasks per lesson/teacher, of which most of the tasks focused on patterns and correlations, comparison and classification, reproduction, transforming and extracting information, and recognition. Nearly all debriefing took place with the class as a whole (plenum), only in one case did students have to check the results on their own with an answering model (18:42 min).

During the debriefing of the tasks, teachers asked on average 12.0 questions during their debriefing. Most of these questions (see Table 4) focused on reproduction, followed by questions focusing on patterns and correlations, recognition, transforming and extracting information, and comparison and classification. A Spearman’s Rho non-parametric analysis was conducted in order to detect correlations between the time spent on debriefing questions and other variables such as the number of tasks set in a lesson, the type of tasks and the number of debriefing questions. The time spent on debriefing and the number of questions during the debriefing were significantly correlated (r_s_ = 0.64, *p* < 0.01), but there was no significant relation between the number of tasks and the number of debriefing questions. Furthermore, the debriefing time correlated significantly with tasks at the level of PHOT and metacognition (r_s_ = 0.51, *p* < 0.05).

**Figure 2:**
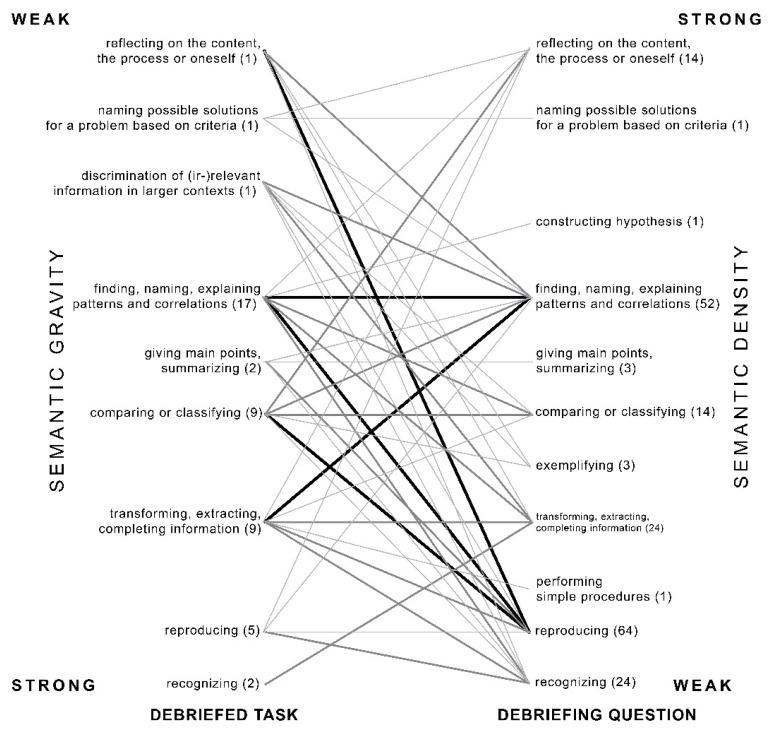
Correlations between tasks and debriefing questions set by the teacher categorized by using the Geography Task Categorization framework based on 47 traceable cases. The numbers (n) indicate the number of tasks or questions. A thick line indicates 11–20 questions, a medium line 4–10 questions and a thin line 1–3 questions.

We were interested in how tasks were discussed during the lessons, and especially to what extent a type of task correlated with a type of question during the debriefing (see Figure 2). Therefore, we looked at 47 tasks, where the teacher was not debriefing two tasks or a whole set of tasks at the same time, and we were able to link 201 debriefing questions to one specific task.

The main focus in task setting of these cases lies on patterns and correlations, and also in the debriefing the main foci become visible: reproducing and again finding, naming and explaining patterns and correlations. Generally, we can see movements towards weaker semantic density as well as towards stronger semantic density. An example for the first is the debriefing of the task at the level of discriminating (ir)relevant information in a complex environment (“What political, cultural, economic and demographic characteristics are typical of Europe? Use your textbook as a source of information”). The teacher moved in the debriefing to the more concrete aspects of transforming, extracting or supplementing information (“Looking at phase four, what can we say about birth rate and death rate?”) and giving examples (“What else is typically Catalan?”). An example for a movement towards strong semantic density is the debriefing of the task of the category extracting information (“How many meters did the floodplain of the Waal rise between 1800 and 2000?”). The students had to obtain this information from a graph. With the debriefing question aiming at correlations (“We have to raise the dikes or …?—teacher waited for an answer from the students which did not come—dig away. What is the harm?”). The teacher tried to connect the information about sedimentation of the river derived from the resource to the discussed lesson topic, to what extent the Dutch rivers should be more regulated or deregulated and get more space. Movements towards stronger semantic density occurred in only 20.9% of the debriefing questions. Movements towards weaker semantic density occurred in approximately two-thirds of the cases (61%).

In 19 of the 47 tasks and 29 of the 201 questions the cognitive processes were aiming at the same category. For example, the task set by the teacher was:

Use atlas maps 163C and D. On the basis of these atlas maps, you can divide China broadly into a center area, a semi-peripheral area and a peripheral area. Indicate which parts of China belong to the center, the semi-periphery and the periphery respectively.

The debriefing question of the same category, comparing or classifying, was “Which areas grow faster?”

**Figure 3:**
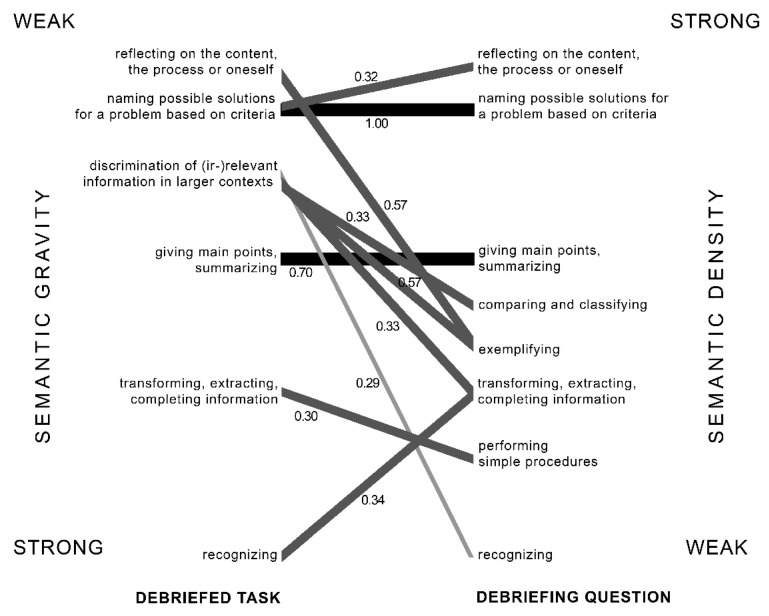
Statistically significant correlations between tasks and debriefing questions set by the teacher categorized by using the Geography Task Categorization framework. The numbers indicate the Spearman’s Rho correlation. A thick line indicates a strong correlation (>0.70), a medium line a moderate correlation (0.30–0.70) and a thin line for a weak correlation (>0.30).

In order to check the relation between task setting and debriefing questions, we calculated Spearman’s Rho instead of the usual Pearson’s correlation, because of our low sample size and in order to avoid assumptions such as normal distribution (see Figure 3). We see that two of the movements towards stronger semantic density were statistically significant, from recognizing towards transforming, extracting or completing information, and from naming possible solutions towards reflecting on the content, the process or oneself. However, six of the statistically significant movements were towards a weaker semantic density, from transforming, extracting and completing information towards performing simple procedures, from discrimination of (irrelevant) information in larger contexts towards recognizing, transforming information, exemplifying, and comparing and classifying, and from reflecting towards exemplifying. In only two categories were correlations with the same type of task and debriefing question statistically significant, e.g., for giving the main points or summarizing and for naming possible solutions.

In addition, we examined if and how teachers related their lessons to the high-stakes exam. In total, 44 times such remarks were made, 26 during the debriefing of questions and 18 times in other parts of the lesson. An example of such a remark is, “If you mention it at 1 then you have to mention something else at 2. The dimensions (cultural, social, economic, natural) come back every time so make sure you know them”, in which the teacher focused on the realization rules for the exam. A one-way ANOVA between groups was performed to compare the number of remarks made about the high-stakes exam and the source of the tasks, e.g., textbook, own materials or former high-stakes exam. There was a statistically significant difference between the groups *F*(2, 46) 4.58, *p* < 0.05. Teachers using textbook tasks referred significantly less to the high-stakes exam (*M* 0.11, *SD* 0.32) than teachers setting former high-stakes exam tasks (*M* 1.1, *SD* 1.62).

## 7. Discussion

We see that two-thirds of the tasks set during the lessons aimed at the use of thinking strategies and one-third at lower order thinking. Only a very few tasks could be categorized as parts of higher thinking or metacognition, and higher order thinking tasks were absent in the observed lessons. In the debriefing we see both movements towards a stronger and, more often, towards weaker semantic density, and in nearly half of the cases questions aimed at lower order thinking. This means that the tasks set during the observed lessons were only partly in line with the intended curriculum; curriculum goals aiming at (parts of) higher order thinking were not met by and large, and in the debriefing students were barely prepared to deal with more complex and condensed information structures.

The results show that the type of tasks, the origin of tasks, and the number of tasks set during the geography lessons were interwoven and cannot be seen as separate from each other. Eighty percent of the tasks set during the observed lessons were derived by the teachers from textbooks. This illustrates the significance of the textbook for the lessons, which indeed makes the textbooks a “materialized curriculum” (see [49]). The analysis of textbook tasks [17] showed that tasks at the level of (parts of) higher order thinking were very scarce, and overreliance on textbook tasks therefore means that curriculum aims related to these levels of thinking might not be achieved sufficiently. Moreover, we see that 9% of the tasks were derived from previous high-stakes exams, and were in three-quarter of the cases already used in the years before in the central examination. As the high-stakes exam focuses dominantly on the use of thinking strategies [14], curriculum aims aspiring to higher levels cannot be reached.

When it comes to the cognitive levels the tasks foster, we see that the distribution of one-third lower order thinking tasks and two-thirds use of thinking strategies, with only a few tasks aiming at parts of higher order thinking, was very similar to the one in the high-stakes exams and especially in the examined textbooks [14] and corresponded with earlier results [1]. The focus clearly lay on the category ‘finding, naming or explaining patterns and correlations’, followed by ‘reproducing’, ‘transforming, extracting or completing information’ and ‘comparing or classifying’. Additionally, here the distribution mirrored the distribution in the textbook, and to some extent also in the high-stakes exam, although there we saw fewer reproduction tasks and a stronger focus on patterns and correlations. When teachers used textbook tasks, they used significantly more lower order thinking tasks compared to tasks from other origins. As the teachers did not select the tasks, but just followed the order as offered by the textbook, this might explain the higher number of lower order thinking tasks. In all three tasks set on the level of parts of higher order thinking and metacognition, these did not come from the textbook nor previous high-stakes exams, but were developed by the teachers themselves (or derived from other sources). Furthermore, in these cases the teacher set only 1–3 tasks. On the other hand, when teachers used more than 11 tasks, this correlated significantly with more lower order thinking and use of thinking strategy tasks. Summarizing, we can state that on the continuum of semantic gravity, the tasks only covered the spectrum between very simple and concrete to medium-complex and abstract knowledge.

Regarding debriefing questions, the number of questions asked correlates positively with the amount of time the teacher spent on debriefing and not with the number of tasks that had been set. If we consider the debriefing questions and the tasks applied in the lessons as routines shaping the legitimate text [4], we see that tasks at the level of (parts of) higher order thinking and metacognition correlated positively with debriefing time. This makes sense, as these tasks are more complex, demand more input from the students, and the discussion of the results here comes more near to what Nichols [41] calls a “debriefing episode”. However, more time spent on debriefing also correlated with tasks in the categories recognizing and reproducing. About 45% of the debriefing questions could be categorized as lower order thinking, and here the debriefing was, in Nichols’ terminology, more like a “question-and-answer episode” [41] (p. 190). Furthermore, we could detect the influence of the high-stakes exam already in the pre-exam year not only in the task setting, but also in the debriefing of tasks. Here the focus lay on how to formulate the legitimate texts which meet the criteria of the realization rules. This might indicate, for the Dutch context, a pre-shadowing effect, which goes further than only school exams [24], and affirms the importance of evaluative rules for the pedagogic practice [14]. 

The main foci in debriefing lay on reproduction and patterns and correlations. However, we see that a certain type of task did not automatically correlate with a debriefing task at the same level, but that the teachers varied between questions stimulating different cognitive processes. These movements show that the path towards more abstract knowledge structures is indeed not a straightforward one, but moving between concrete and abstract [8], and that teachers in this way tried to support mastering semantic gravity [4]. However, the movements were mostly limited to the levels of lower order thinking and use of thinking strategies. Furthermore, movements towards stronger semantic density occurred in only one-fifth of the debriefing questions, of which two were statistically significant. Movements towards weaker semantic density occurred in approximately two-thirds of the cases, and five of these relations were statistically significant. With respect to curriculum aims, we can state that debriefing questions barely addressed (parts of) higher order thinking, and addressed metacognitive aspects to some extent.

With respect to the results presented in this paper, we have to make two caveats. First, although all lessons were recorded in a period amply before the period of central examination, only one lesson topic would not be assessed later in the high-stakes exam. This means that if more lessons about topics which were not examined in the central examination would have been observed, tasks set during the lesson might have aimed more at (parts of) higher order thinking. However, the analysis of textbook tasks about these topics examined only in a school exam shows that tasks aiming at (parts of) higher order thinking barely occurred in the textbooks [16,17], on which teachers seemed to rely so heavily. Second, the lessons observed were limited to twenty-three teachers in only the southern part of the country at the highest level of upper secondary education. This means that the observations are not representative for all Dutch teachers, and that regional differences as well as differences between other types of upper secondary education might occur.

## 8. Conclusions

The aim of this research was to answer the question to what extent tasks in Dutch geography lessons are in line with the intended curriculum. We focused on the tasks set and questions used during the debriefing of the tasks during twenty-three video-recorded lessons. Teachers face an ambiguity between the intended curriculum on one side, and high-stakes exams and textbooks on the other side. The intended curriculum focuses on systematic knowledge achieved by the use of thinking strategies, but also aims at (parts of) higher order thinking and metacognition, but this last aspect is barely taken into consideration by high-stakes exams and textbooks.

The results show that teachers recontextualized geographical knowledge in their task setting and debriefing in line with the high-stakes exam, and that textbooks played a dominant role. This means that the evaluative rules of the pedagogic device, as they materialize in the high-stakes exams, influence the teaching in upper secondary education, and not only shortly before that examination takes place, which confirms a trend of ‘teaching to the test’ being observed for Dutch upper secondary education in general [49]. Teachers use mainly textbook tasks, but these tasks are not carefully selected; teachers rather follow the number and order of tasks as offered by the textbooks. This implies that textbooks de facto become the lesson [50] or curriculum [51]. Although Dutch textbook publishers state that they take the curriculum aims into account when developing textbooks, this counts more for factual and conceptual knowledge than for cognitive processes at the level of (parts of) higher order thinking, and tasks are constructed alike in the high-stakes exams [1]. Thus, an overreliance on the textbook, complemented with examples from previous high-stakes exams, in the recontextualization process does not allow the teacher to meet the intended aims of the curriculum on the level of (parts of) higher order thinking and to adapt their teaching to the specific learning needs of their students. By doing so, they become executors of textbooks. Sequentially, it has to be considered whether the central examination has to be adapted towards (parts of) higher order thinking, as it was once intended [52,53]. Bernstein [15] suggests a change in classification and framing would change the interactional practice. In concrete terms, this means that a change of the high-stakes exam in aspect of realization rules and legitimate text would lead to a change of tasks in teaching practice and textbooks. Furthermore, textbook publishers and authors, as tasks are a key characteristic for the quality of textbooks [54,55], should reconsider their supply of tasks in the number, cognitive processes and sequencing towards a higher share of tasks aiming at (parts of) higher order thinking and metacognition. Finally, curriculum regulations should provide more leeway for teachers to act as curriculum makers, and teachers must be prepared for this role in their initial teacher training [49].

Thus, in respect of semantic gravity and density, task setting and debriefing is limited to the bottom and the middle part in the Geography Task Categorization Framework, which means that meanings are more concrete and less abstract, and the contexts are presented as less complex. The highest level to be achieved in these lessons can be characterized as systematic knowledge achieved by the use of thinking strategies. There are barely tasks and debriefing questions at the level of (parts of) higher order thinking. Mostly there is “one correct answer”, and limited chance to discuss a variety of possibilities, as only seldom does a “discursive gap” open. However, between reproducing facts and concepts and the use of thinking strategies, teachers move in their debriefing between concrete and abstract, which is considered to be key for cumulative learning [4,8]. An important question here is to what extent the debriefing of teachers is a conscious act in the recontextualization process, as the task selection itself indicates otherwise. Furthermore, we know that the debriefing of tasks is considered to be a challenge for teachers [41], and they might need support in this matter, whether it be by materials from the textbook publishers, by courses in continuous professional development, or during their education. Furthermore, a change towards an inquiry approach and starting by confronting students with the inquiry question, might foster debriefing towards a stronger semantic density [56]. However, the debriefing of tasks, just like task setting itself, perhaps does not reflect the teachers’ knowledge and capabilities [57], but might be influenced by the evaluative rules of the curriculum context, in which the form of high-stakes exams play a key role. It also has to be explored if similar results occur in the teaching of other subjects.

## Figures and Tables

**Table 1:** Average lesson time spent on tasks.

Average Lesson Time in Minutes	Average Effective Lesson Time in Minutes	Average Time Spent on Making Tasks in % of the Effective Lesson Time	Average Time spent on Debriefing Time in % of the Effective Lesson Time	Average Time spent Simultaneously on Making and Debriefing Task in % of the Effective Lesson Time
46:17	42:09	27%	22%	25%

**Table 2:** Tasks set during the lessons categorized according to the Geography Task Categorization Framework.

Number of Tasks	Percentage of Tasks Set during Lessons According the Geography Task Categorization Framework												
	Lower Order Thinking (Lot)				Use of Thinking Strategies (Uts)						Parts of Higher Order Thinking (Phot)		Metacognition
Type of task ^1^	1	2	3	4	5	6	7	8	9	10	11	15	19
238	1.3	7.6	21.4	1.7	21.0	0.8	11.3	0.4	32.4	0.8	0.4	0.4	0.4

^1^ 1 = Gathering or naming information without any further task; 2 = Recognizing; 3 = Reproducing; 4 = Performing (simple procedures); 5 = Transforming, extracting or completing information; 6 = Exemplifying; 7 = Comparing or classifying; 8 = Giving the main points or summarizing; 9 = Finding, naming or explaining patterns and correlations; 10 = Constructing hypotheses or formulating enquiry questions; 11 = Discrimination of (ir-)relevant information in larger contexts; 15 = Naming possible solutions for a problem based on criteria; 19 = Reflecting on the content, the process or oneself.

**Table 3:** Debriefed tasks categorized according to the Geography Task Categorization Framework.

Number of Tasks	Percentage of Tasks during Debriefing According the Geography Task Categorization Framework									
	Lower Order Thinking (Lot)			Use of Thinking Strategies (Uts)				Parts of Higher Order Thinking (Phot)		Metacognition
Type of task ^1^	2	3	4	5	7	8	9	11	15	19
96	8.3	16.7	2.1	10.4	20.1	2.1	35.4	1.0	1.0	2.0

^1^ 2 = Recognizing; 3 = Reproducing; 4 = Performing (simple procedures); 5 = Transforming, extracting or completing information; 7 = Comparing or classifying; 8 = Giving the main points or summarizing; 9 = Finding, naming or explaining patterns and correlations; 11 = Discrimination of (ir-)relevant information in larger contexts; 15 = Naming possible solutions for a problem based on criteria; 19 = Reflecting on the content, the process or oneself.

**Table 4:** Questions asked during the debriefing of tasks categorized according to the Geography Task Categorization Framework.

Number of Questions	Percentage of Questions during Debriefing of Tasks According the Geography Task Categorization Framework											
	Lower Order Thinking (Lot)			Use of Thinking Strategies (Uts)						Parts of Higher Order Thinking (Phot)		Metacognition
Type of question ^1^	2	3	4	5	6	7	8	9	10	11	15	19
210	12.9	31.9	0.5	11.9	1.9	6.7	1.4	25.7	0.5	0.5	0.5	7.6

^1^ 2 = Recognizing; 3 = Reproducing; 4 = Performing (simple procedures); 5 = Transforming, extracting or completing information; 6 = Exemplifying; 7 = Comparing or classifying; 8 = Giving the main points or summarizing; 9 = Finding, naming or explaining patterns and correlations; 10 = Constructing hypotheses or formulating enquiry questions; 11 = Discrimination of (ir-)relevant information in larger contexts; 15 = Naming possible solutions for a problem based on criteria; 19 = Reflecting on the content, the process or oneself.

## Data Availability

The data presented in this study are available on request from the corresponding author. The data are not publicly available due to privacy reasons.
