# Correction: Krause et al. Higher Order Thinking by Setting and Debriefing Tasks in Dutch Geography Lessons. *Eur. J. Investig. Health Psychol. Educ.* 2022, *12*, 11–27

**DOI:** 10.3390/ejihpe12100105

**Published:** 2022-10-09

**Authors:** Uwe Krause, Tine Béneker, Jan van Tartwijk

**Affiliations:** 1 Faculty of Geosciences, Utrecht University, 3584 CB Utrecht, The Netherlands; 2 Department of Geography Education, Fontys University of Applied Sciences Tilburg, 5022 DM Tilburg, The Netherlands; 3 Faculty of Social and Behavioural Sciences, Utrecht University, 3584 CS Utrecht, The Netherlands

## Incorrect Last Name in Citation

In the original publication [1], there was a mistake in the **Citation**. For the author’s name “Jan van Tartwijk-, Tartwijk, J.v. should be van Tartwijk, J.

## Error in Figure

In the original publication [1], there was a mistake in **Figure 1,** as published. **For “semantic gravity”, “strong” and “weak” should be reversed.** The corrected **Figure 1** appears below.

The authors state that the scientific conclusions are unaffected. This correction was approved by the Academic Editor. The original publication has also been updated.

## Figures and Tables

**Figure 1:**
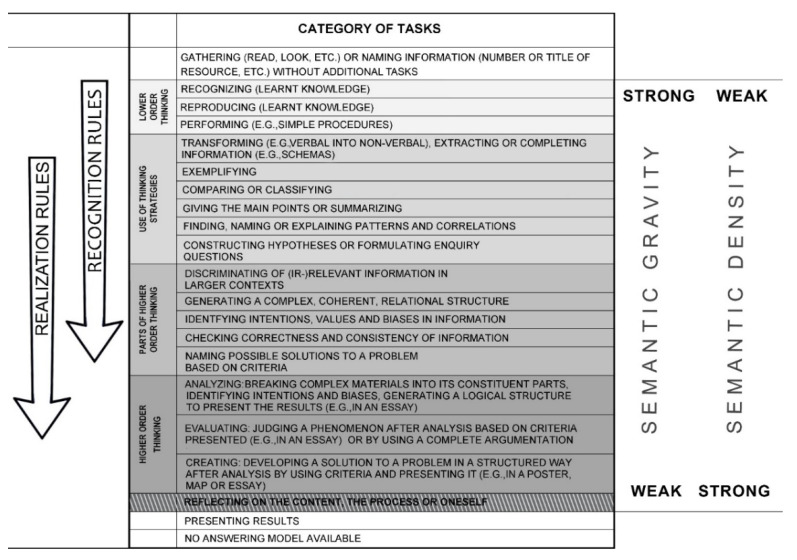
Geography Task Categorization Framework—the arrows indicate the increase of importance of recognition and realization rules (adapted from [16])—including the concept of semantic gravity and density of Maton [4]).
